# A Case of Extensive Cerebral Softening1Read before the Physicians’ League, April 6, 1896.

**Published:** 1896-06

**Authors:** Helene Kuhlmann

**Affiliations:** Assistant Physician, Buffalo State Hospital


### A CASE OF EXTENSIVE CEREBRAL SOFTENING.[1]

By HELENE KUHLMANN, M. D.,
Assistant Physician, Buffalo State Hospital.

THE reasons for reporting the following case are the extent of the lesion and the peculiarity of the manner of invasion of the different areas of the brain, which was very systematic and showed several points of interest:

Mrs. A. D., age 53; widow; nativity, England; number of children unascertained; no insanity in family as far as known; habits good; no history of syphilis.

She was admitted to the Buffalo State Hospital on January 5, 1894, suffering from terminal dementia of about thirteen years' duration, the history accompanying her being very scanty and incomplete. Physical condition on entering was poor; she was feeble, anemic, and was suffering from general atheroma. Heart was rapid and irregular; there was no murmur; pulse tense; tongue deviated slightly to the right; pupils were dilated and equal; gait normal, no paralysis of limbs.

She was controlled by delusions of grandeur; believed that she was Queen Victoria, that she possessed unlimited power, could command people to be executed; mistook the identity of physicians, nurses and patients; was often noisy, profane and obscene, and assumed a threatening attitude if anyone approached her. Had evidently marked hallucinations of hearing.

Her general condition remained unchanged until the morning of November 20, 1895, when she had a convulsion, followed by temporary loss of consciousness. The facts as related by the nurse are as follows: Patient ate her breakfast as usual at 7 A. M., showing nothing abnormal. A few minutes before 8 she lay down on the couch, being somewhat pale, and at 8 she had a convulsion, unaccompanied by frothing at the mouth. Unconsciousness lasted about 20 minutes. On examining her one hour later, the right arm was found to be paralysed; no other focal symptoms.

November 21st—Still unable to use right arm; speaks plainly; is hilarious and emotional; pupils are normal; has repeatedly gotten up out of bed and walked around the room; swallows well.

November 22d—This morning entire right side of the body is paralysed. Lines of right side of the face are obliterated; corner of the mouth drawn to left; ptosis of right lid; in addition to paralysis, anesthesia of right arm and leg; is very stupid; speech indistinct. Patient had been unusually quiet during the preceding night, and there had been no report or evidences of further convulsions. The paralysis gradually deepened during the succeeding days. On November 26th,

---

1. Read before the Physicians' League, April 6, 1896.

patient was utterly helpless, unable to move, swallowing with great difficulty; there was complete aphasia and paralysis of the sphincters of bladder and rectum. She remained in this condition until death occurred on the morning of the 28th. The autopsy was performed twenty-five hours after death, and was confined to examination of the brain. The calvarium was found slightly adherent; dura mater congested; no effusion. Arteries everywhere atheromatous, especially the posterior cerebral on the left side and the posterior communicating. Patches of atheroma irregularly distributed throughout the circle of Willis. The middle cerebral on the left side was extremely atheromatous, the patches being especially marked at the bifurcation of the fissure of Sylvius, where the artery divides into its principal branches. At this point a clot was found, about the size of a pea, completely filling the lumen of the vessel. It was dark red in color and partially organised. The white matter of the left side showed extensive white softening, especially marked in the temporo-sphenoidal lobe, the third frontal convolution, the occipital lobe and the posterior inferior parietal lobe. The brain tissue was opalescent and showed an absence of the puncta vasculorum, which gave the brain a marble-like appearance. The gray matter of the left side had undergone so much disintegration that its thickness could not be accurately ascertained, but it was considerably decreased. The right hemisphere was comparatively normal, with the exception of the atheroma, which was not as marked as on the left side.

The points of interest in the case are, in the first place, the manner of onset, which on the first day looked like that of embolism and on the third day certainly gave the impression of cerebral hemorrhage, considering the complete hemiplegia. It shows how difficult it often is to differentiate accurately between hemorrhage, embolism and thrombosis. On considering the symptom complex further, we find that it corresponds exactly to the nature of the lesion. Softening evidently first occurred in the motor area, supplied by the second and third branches of the middle cerebral artery and the first center affected was that of the arm. A day elapsed before the softening of the leg and face centers had become complete, and about the same time softening of Broca's convolution, supplied by the first branch, the inferior frontal, had reached its completion. The softening of the occipital and temporo-sphenoidal lobes shows that the blood supply of the posterior cerebral was very insufficient, and we have seen that it was in a state of advanced atheroma. The degeneration of the gray matter was, of course, produced by the cutting off of the supply of the nutrient arteries.

Whether the patient's mental condition on admission—the dementia—had been caused by an embolus or thrombus at some previous time, as might be indicated by deviation of the tongue, could not be ascertained, but does not seem at all improbable.

